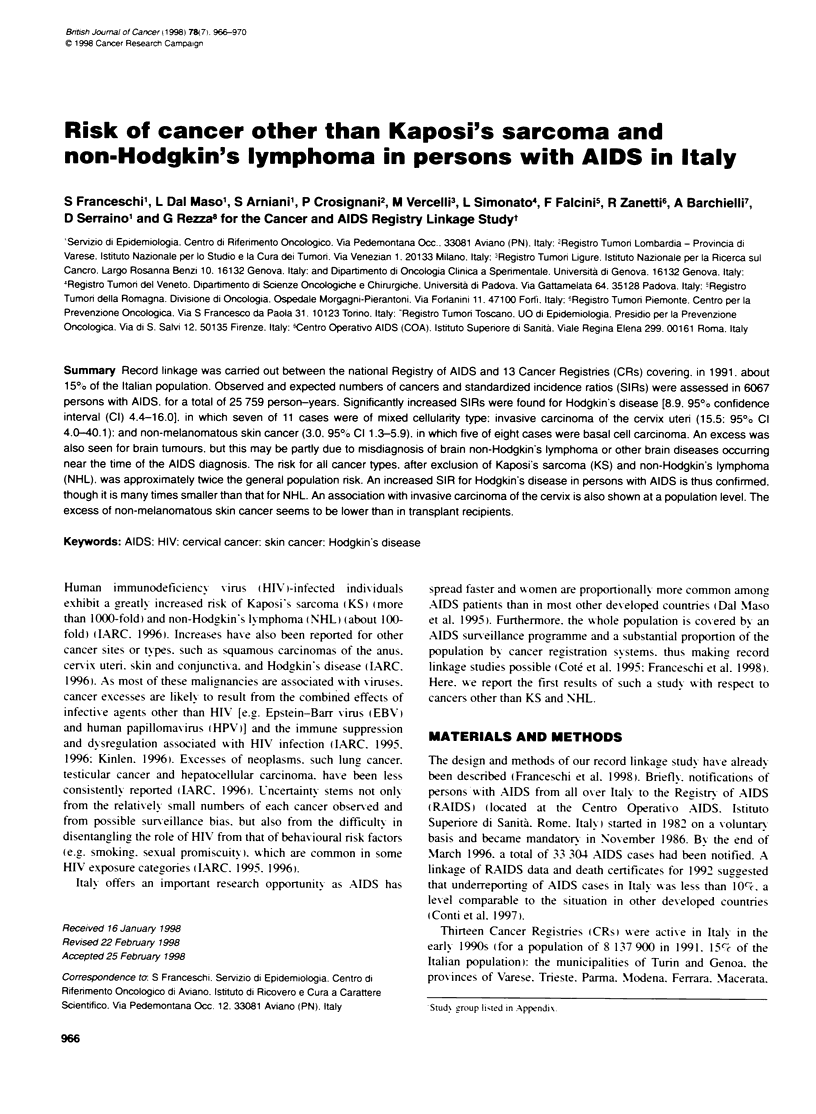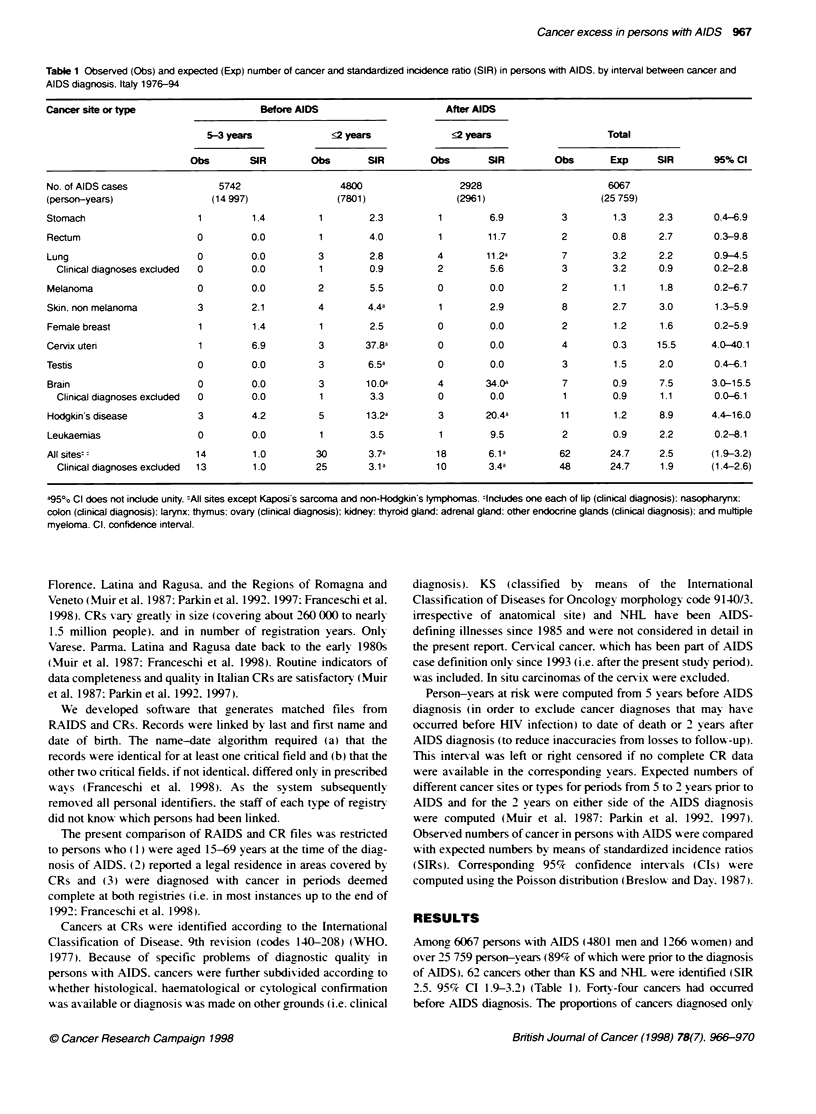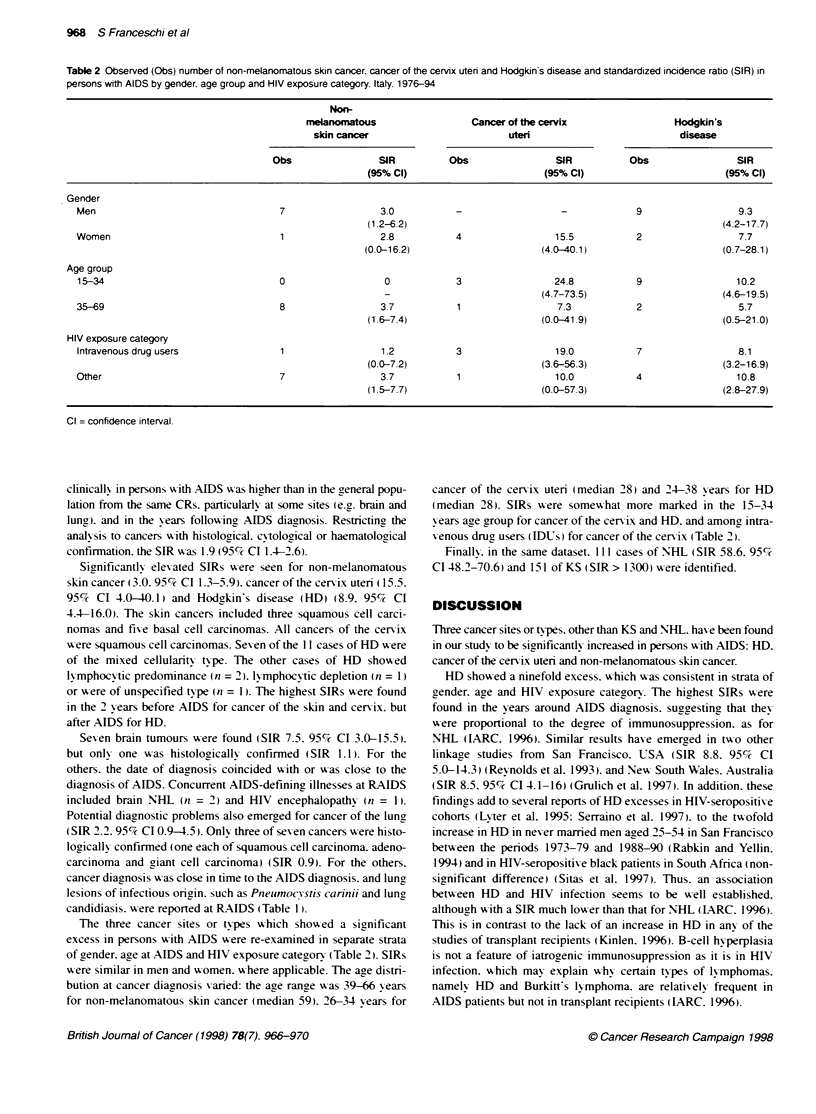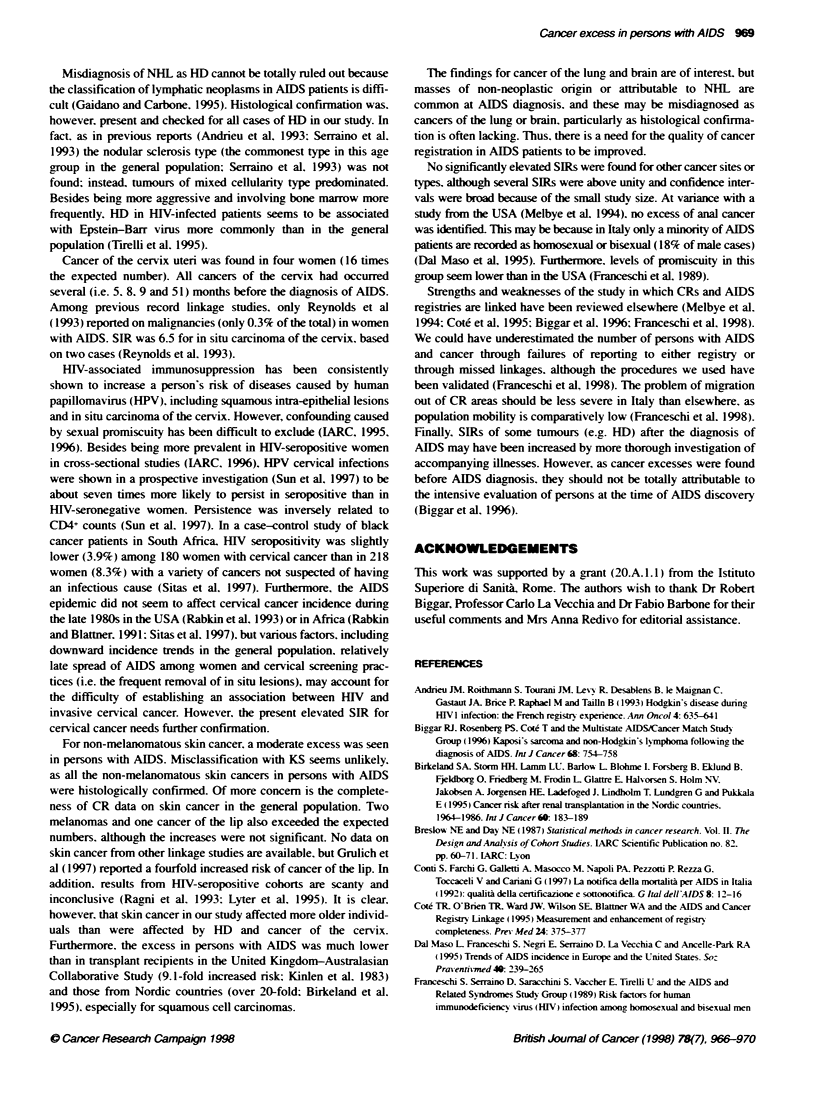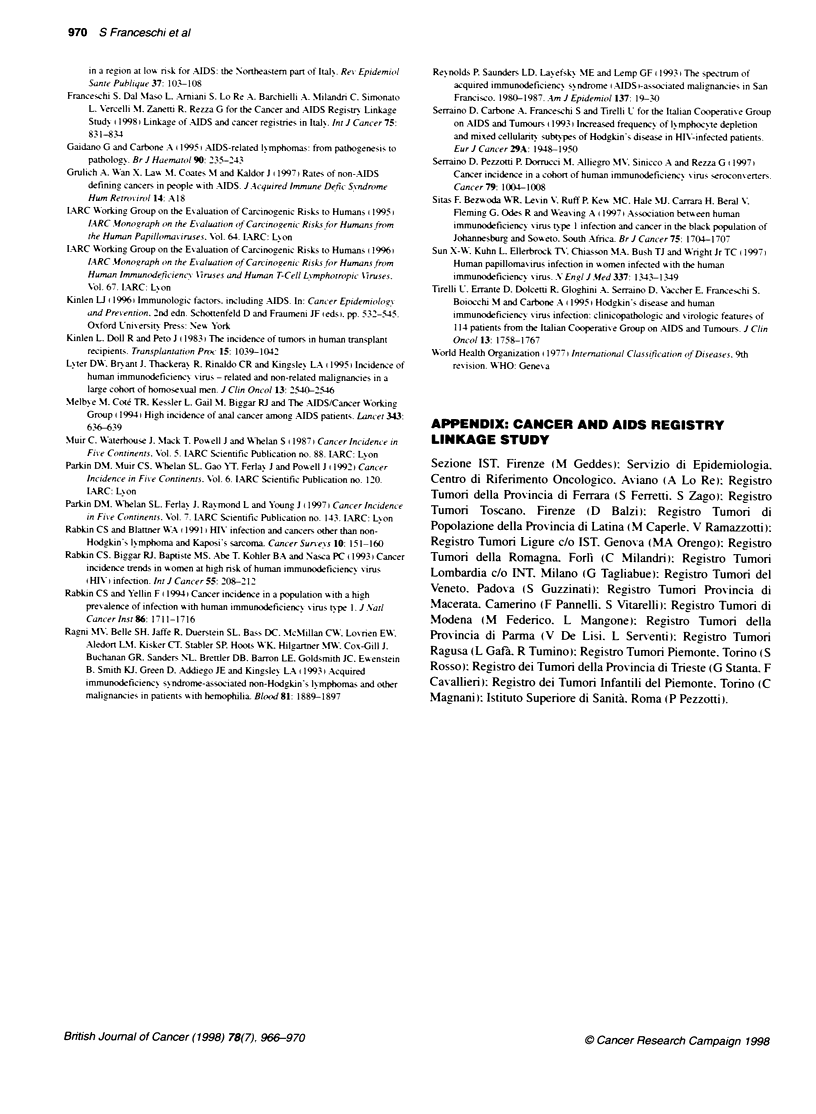# Risk of cancer other than Kaposi's sarcoma and non-Hodgkin's lymphoma in persons with AIDS in Italy. Cancer and AIDS Registry Linkage Study.

**DOI:** 10.1038/bjc.1998.610

**Published:** 1998-10

**Authors:** S. Franceschi, L. Dal Maso, S. Arniani, P. Crosignani, M. Vercelli, L. Simonato, F. Falcini, R. Zanetti, A. Barchielli, D. Serraino, G. Rezza

**Affiliations:** Servizio di Epidemiologia, Centro di Riferimento Oncologico, Aviano (PN), Italy.

## Abstract

Record linkage was carried out between the national Registry of AIDS and 13 Cancer Registries (CRs) covering, in 1991, about 15% of the Italian population. Observed and expected numbers of cancers and standardized incidence ratios (SIRs) were assessed in 6067 persons with AIDS, for a total of 25,759 person-years. Significantly increased SIRs were found for Hodgkin's disease [8.9, 95% confidence interval (CI) 4.4-16.0], in which seven of 11 cases were of mixed cellularity type; invasive carcinoma of the cervix uteri (15.5; 95% CI 4.0-40.1); and non-melanomatous skin cancer (3.0, 95% CI 1.3-5.9), in which five of eight cases were basal cell carcinoma. An excess was also seen for brain tumours, but this may be partly due to misdiagnosis of brain non-Hodgkin's lymphoma or other brain diseases occurring near the time of the AIDS diagnosis. The risk for all cancer types, after exclusion of Kaposi's sarcoma (KS) and non-Hodgkin's lymphoma (NHL), was approximately twice the general population risk. An increased SIR for Hodgkin's disease in persons with AIDS is thus confirmed, though it is many times smaller than that for NHL. An association with invasive carcinoma of the cervix is also shown at a population level. The excess of non-melanomatous skin cancer seems to be lower than in transplant recipients.


British Journal of Cancer (1998) 78(7), 966–970
© 1998 Cancer Research Campaign

### Risk of cancer other than Kaposi's sarcoma and non-Hodgkin's lymphoma in persons with AIDS in Italy

**S Franceschi[1], L Dal Maso[1], S Arniani[1], P Crosignani[2], M Vercelli[3], L Simonato[4], F Falcini[5], R Zanetti[6], A Barchielli[7], D Serraino[1] and G Rezza[8] for the Cancer and AIDS Registry Linkage Study†**

[1]Servizio di Epidemiologia. Centro di Riferimento Oncologico. Via Pedemontana Occ.. 33081 Aviano (PN). Italy: [2]Registro Tumori Lombardia – Provincia di Varese. Istituto Nazionale per lo Studio e la Cura dei Tumori. Via Venezian 1. 20133 Milano. Italy: [3]Registro Tumori Ligure. Istituto Nazionale per la Ricerca sul Cancro. Largo Rosanna Benzi 10. 16132 Genova. Italy: and Dipartimento di Oncologia Clinica a Sperimentale. Università di Genova. 16132 Genova. Italy: [4]Registro Tumori del Veneto. Dipartimento di Scienze Oncologiche e Chirurgiche. Università di Padova. Via Gattamelata 64. 35128 Padova. Italy: [5]Registro Tumori della Romagna. Divisione di Oncologia. Ospedale Morgagni-Pierantoni. Via Forlanini 11. 47100 Forlì. Italy: [6]Registro Tumori Piemonte. Centro per la Prevenzione Oncologica. Via S Francesco da Paola 31. 10123 Torino. Italy: [7]Registro Tumori Toscano. UO di Epidemiologia. Presidio per la Prevenzione Oncologica. Via di S. Salvi 12. 50135 Firenze. Italy: [8]Centro Operativo AIDS (COA). Istituto Superiore di Sanità. Viale Regina Elena 299. 00161 Roma. Italy

**Summary** Record linkage was carried out between the national Registry of AIDS and 13 Cancer Registries (CRs) covering. in 1991. about 15% of the Italian population. Observed and expected numbers of cancers and standardized incidence ratios (SIRs) were assessed in 6067 persons with AIDS. for a total of 25 759 person–years. Significantly increased SIRs were found for Hodgkin's disease [8.9. 95% confidence interval (CI) 4.4–16.0]. in which seven of 11 cases were of mixed cellularity type: invasive carcinoma of the cervix uteri (15.5: 95% CI 4.0–40.1): and non-melanomatous skin cancer (3.0. 95% CI 1.3–5.9). in which five of eight cases were basal cell carcinoma. An excess was also seen for brain tumours. but this may be partly due to misdiagnosis of brain non-Hodgkin's lymphoma or other brain diseases occurring near the time of the AIDS diagnosis. The risk for all cancer types. after exclusion of Kaposi's sarcoma (KS) and non-Hodgkin's lymphoma (NHL). was approximately twice the general population risk. An increased SIR for Hodgkin's disease in persons with AIDS is thus confirmed. though it is many times smaller than that for NHL. An association with invasive carcinoma of the cervix is also shown at a population level. The excess of non-melanomatous skin cancer seems to be lower than in transplant recipients.

**Keywords:** AIDS: HIV: cervical cancer: skin cancer: Hodgkin's disease

Human immunodeficiency virus (HIV)-infected individuals exhibit a greatly increased risk of Kaposi's sarcoma (KS) (more than 1000-fold) and non-Hodgkin's lymphoma (NHL) (about 100-fold) (IARC. 1996). Increases have also been reported for other cancer sites or types. such as squamous carcinomas of the anus. cervix uteri. skin and conjunctiva. and Hodgkin's disease (IARC. 1996). As most of these malignancies are associated with viruses. cancer excesses are likely to result from the combined effects of infective agents other than HIV [e.g. Epstein–Barr virus (EBV) and human papillomavirus (HPV)] and the immune suppression and dysregulation associated with HIV infection (IARC. 1995. 1996: Kinlen. 1996). Excesses of neoplasms. such lung cancer. testicular cancer and hepatocellular carcinoma. have been less consistently reported (IARC. 1996). Uncertainty stems not only from the relatively small numbers of each cancer observed and from possible surveillance bias. but also from the difficulty in disentangling the role of HIV from that of behavioural risk factors (e.g. smoking. sexual promiscuity). which are common in some HIV exposure categories (IARC. 1995. 1996).

Italy offers an important research opportunity as AIDS has spread faster and women are proportionally more common among AIDS patients than in most other developed countries (Dal Maso et al. 1995). Furthermore. the whole population is covered by an AIDS surveillance programme and a substantial proportion of the population by cancer registration systems. thus making record linkage studies possible (Coté et al. 1995: Franceschi et al. 1998). Here. we report the first results of such a study with respect to cancers other than KS and NHL.

### MATERIALS AND METHODS

The design and methods of our record linkage study have already been described (Franceschi et al. 1998). Briefly. notifications of persons with AIDS from all over Italy to the Registry of AIDS (RAIDS) (located at the Centro Operativo AIDS. Istituto Superiore di Sanità. Rome. Italy) started in 1982 on a voluntary basis and became mandatory in November 1986. By the end of March 1996. a total of 33 304 AIDS cases had been notified. A linkage of RAIDS data and death certificates for 1992 suggested that underreporting of AIDS cases in Italy was less than 10%. a level comparable to the situation in other developed countries (Conti et al. 1997).

Thirteen Cancer Registries (CRs) were active in Italy in the early 1990s (for a population of 8 137 900 in 1991. 15% of the Italian population): the municipalities of Turin and Genoa. the provinces of Varese. Trieste. Parma. Modena. Ferrara. Macerata.

†Study group listed in Appendix.

*Received 16 January 1998*
*Revised 22 February 1998*
*Accepted 25 February 1998*

*Correspondence to:* S Franceschi. Servizio di Epidemiologia. Centro di Riferimento Oncologico di Aviano. Istituto di Ricovero e Cura a Carattere Scientifico. Via Pedemontana Occ. 12. 33081 Aviano (PN). Italy

**Table 1** Observed (Obs) and expected (Exp) number of cancer and standardized incidence ratio (SIR) in persons with AIDS, by interval between cancer and AIDS diagnosis, Italy 1976–94

| Cancer site or type | Before AIDS | | | | After AIDS | | | | | | |
|---|---|---|---|---|---|---|---|---|---|---|---|
| | 5–3 years | | ≤2 years | | ≤2 years | | Total | | | | |
| | Obs | SIR | Obs | SIR | Obs | SIR | Obs | Exp | SIR | 95% CI | |
| No. of AIDS cases (person–years) | 5742 (14 997) | | 4800 (7801) | | 2928 (2961) | | | 6067 (25 759) | | | |
| Stomach | 1 | 1.4 | 1 | 2.3 | 1 | 6.9 | 3 | 1.3 | 2.3 | 0.4–6.9 | |
| Rectum | 0 | 0.0 | 1 | 4.0 | 1 | 11.7 | 2 | 0.8 | 2.7 | 0.3–9.8 | |
| Lung | 0 | 0.0 | 3 | 2.8 | 4 | 11.2[a] | 7 | 3.2 | 2.2 | 0.9–4.5 | |
| Clinical diagnoses excluded | 0 | 0.0 | 1 | 0.9 | 2 | 5.6 | 3 | 3.2 | 0.9 | 0.2–2.8 | |
| Melanoma | 0 | 0.0 | 2 | 5.5 | 0 | 0.0 | 2 | 1.1 | 1.8 | 0.2–6.7 | |
| Skin, non melanoma | 3 | 2.1 | 4 | 4.4[a] | 1 | 2.9 | 8 | 2.7 | 3.0 | 1.3–5.9 | |
| Female breast | 1 | 1.4 | 1 | 2.5 | 0 | 0.0 | 2 | 1.2 | 1.6 | 0.2–5.9 | |
| Cervix uteri | 1 | 6.9 | 3 | 37.8[a] | 0 | 0.0 | 4 | 0.3 | 15.5 | 4.0–40.1 | |
| Testis | 0 | 0.0 | 3 | 6.5[a] | 0 | 0.0 | 3 | 1.5 | 2.0 | 0.4–6.1 | |
| Brain | 0 | 0.0 | 3 | 10.0[a] | 4 | 34.0[a] | 7 | 0.9 | 7.5 | 3.0–15.5 | |
| Clinical diagnoses excluded | 0 | 0.0 | 1 | 3.3 | 0 | 0.0 | 1 | 0.9 | 1.1 | 0.0–6.1 | |
| Hodgkin's disease | 3 | 4.2 | 5 | 13.2[a] | 3 | 20.4[a] | 11 | 1.2 | 8.9 | 4.4–16.0 | |
| Leukaemias | 0 | 0.0 | 1 | 3.5 | 1 | 9.5 | 2 | 0.9 | 2.2 | 0.2–8.1 | |
| All sites[b,c] | 14 | 1.0 | 30 | 3.7[a] | 18 | 6.1[a] | 62 | 24.7 | 2.5 | (1.9–3.2) | |
| Clinical diagnoses excluded | 13 | 1.0 | 25 | 3.1[a] | 10 | 3.4[a] | 48 | 24.7 | 1.9 | (1.4–2.6) | |

[a]95% CI does not include unity. [b]All sites except Kaposi's sarcoma and non-Hodgkin's lymphomas. [c]Includes one each of lip (clinical diagnosis); nasopharynx; colon (clinical diagnosis); larynx; thymus; ovary (clinical diagnosis); kidney; thyroid gland; adrenal gland; other endocrine glands (clinical diagnosis); and multiple myeloma. CI, confidence interval.

Florence, Latina and Ragusa, and the Regions of Romagna and Veneto (Muir et al. 1987; Parkin et al. 1992, 1997; Franceschi et al. 1998). CRs vary greatly in size (covering about 260 000 to nearly 1.5 million people), and in number of registration years. Only Varese, Parma, Latina and Ragusa date back to the early 1980s (Muir et al. 1987; Franceschi et al. 1998). Routine indicators of data completeness and quality in Italian CRs are satisfactory (Muir et al. 1987; Parkin et al. 1992, 1997).

We developed software that generates matched files from RAIDS and CRs. Records were linked by last and first name and date of birth. The name–date algorithm required (a) that the records were identical for at least one critical field and (b) that the other two critical fields, if not identical, differed only in prescribed ways (Franceschi et al. 1998). As the system subsequently removed all personal identifiers, the staff of each type of registry did not know which persons had been linked.

The present comparison of RAIDS and CR files was restricted to persons who (1) were aged 15–69 years at the time of the diagnosis of AIDS, (2) reported a legal residence in areas covered by CRs and (3) were diagnosed with cancer in periods deemed complete at both registries (i.e. in most instances up to the end of 1992; Franceschi et al. 1998).

Cancers at CRs were identified according to the International Classification of Disease, 9th revision (codes 140–208) (WHO, 1977). Because of specific problems of diagnostic quality in persons with AIDS, cancers were further subdivided according to whether histological, haematological or cytological confirmation was available or diagnosis was made on other grounds (i.e. clinical diagnosis). KS (classified by means of the International Classification of Diseases for Oncology morphology code 9140/3, irrespective of anatomical site) and NHL have been AIDS-defining illnesses since 1985 and were not considered in detail in the present report. Cervical cancer, which has been part of AIDS case definition only since 1993 (i.e. after the present study period), was included. In situ carcinomas of the cervix were excluded.

Person–years at risk were computed from 5 years before AIDS diagnosis (in order to exclude cancer diagnoses that may have occurred before HIV infection) to date of death or 2 years after AIDS diagnosis (to reduce inaccuracies from losses to follow-up). This interval was left or right censored if no complete CR data were available in the corresponding years. Expected numbers of different cancer sites or types for periods from 5 to 2 years prior to AIDS and for the 2 years on either side of the AIDS diagnosis were computed (Muir et al. 1987; Parkin et al. 1992, 1997). Observed numbers of cancer in persons with AIDS were compared with expected numbers by means of standardized incidence ratios (SIRs). Corresponding 95% confidence intervals (CIs) were computed using the Poisson distribution (Breslow and Day, 1987).

### RESULTS

Among 6067 persons with AIDS (4801 men and 1266 women) and over 25 759 person–years (89% of which were prior to the diagnosis of AIDS), 62 cancers other than KS and NHL were identified (SIR 2.5, 95% CI 1.9–3.2) (Table 1). Forty-four cancers had occurred before AIDS diagnosis. The proportions of cancers diagnosed only

© Cancer Research Campaign 1998

**Table 2** Observed (Obs) number of non-melanomatous skin cancer, cancer of the cervix uteri and Hodgkin's disease and standardized incidence ratio (SIR) in persons with AIDS by gender, age group and HIV exposure category, Italy, 1976–94

| | Non-melanomatous skin cancer | | Cancer of the cervix uteri | | Hodgkin's disease | |
|---|---|---|---|---|---|---|
| | Obs | SIR (95% CI) | Obs | SIR (95% CI) | Obs | SIR (95% CI) |
| Gender | | | | | | |
| Men | 7 | 3.0 (1.2–6.2) | – | – | 9 | 9.3 (4.2–17.7) |
| Women | 1 | 2.8 (0.0–16.2) | 4 | 15.5 (4.0–40.1) | 2 | 7.7 (0.7–28.1) |
| Age group | | | | | | |
| 15–34 | 0 | 0 – | 3 | 24.8 (4.7–73.5) | 9 | 10.2 (4.6–19.5) |
| 35–69 | 8 | 3.7 (1.6–7.4) | 1 | 7.3 (0.0–41.9) | 2 | 5.7 (0.5–21.0) |
| HIV exposure category | | | | | | |
| Intravenous drug users | 1 | 1.2 (0.0–7.2) | 3 | 19.0 (3.6–56.3) | 7 | 8.1 (3.2–16.9) |
| Other | 7 | 3.7 (1.5–7.7) | 1 | 10.0 (0.0–57.3) | 4 | 10.8 (2.8–27.9) |

CI = confidence interval.

clinically in persons with AIDS was higher than in the general population from the same CRs, particularly at some sites (e.g. brain and lung), and in the years following AIDS diagnosis. Restricting the analysis to cancers with histological, cytological or haematological confirmation, the SIR was 1.9 (95% CI 1.4–2.6).

Significantly elevated SIRs were seen for non-melanomatous skin cancer (3.0, 95% CI 1.3–5.9), cancer of the cervix uteri (15.5, 95% CI 4.0–40.1) and Hodgkin's disease (HD) (8.9, 95% CI 4.4–16.0). The skin cancers included three squamous cell carcinomas and five basal cell carcinomas. All cancers of the cervix were squamous cell carcinomas. Seven of the 11 cases of HD were of the mixed cellularity type. The other cases of HD showed lymphocytic predominance ($n$ = 2), lymphocytic depletion ($n$ = 1) or were of unspecified type ($n$ = 1). The highest SIRs were found in the 2 years before AIDS for cancer of the skin and cervix, but after AIDS for HD.

Seven brain tumours were found (SIR 7.5, 95% CI 3.0–15.5), but only one was histologically confirmed (SIR 1.1). For the others, the date of diagnosis coincided with or was close to the diagnosis of AIDS. Concurrent AIDS-defining illnesses at RAIDS included brain NHL ($n$ = 2) and HIV encephalopathy ($n$ = 1). Potential diagnostic problems also emerged for cancer of the lung (SIR 2.2, 95% CI 0.9–4.5). Only three of seven cancers were histologically confirmed (one each of squamous cell carcinoma, adenocarcinoma and giant cell carcinoma) (SIR 0.9). For the others, cancer diagnosis was close in time to the AIDS diagnosis, and lung lesions of infectious origin, such as *Pneumocystis carinii* and lung candidiasis, were reported at RAIDS (Table 1).

The three cancer sites or types which showed a significant excess in persons with AIDS were re-examined in separate strata of gender, age at AIDS and HIV exposure category (Table 2). SIRs were similar in men and women, where applicable. The age distribution at cancer diagnosis varied: the age range was 39–66 years for non-melanomatous skin cancer (median 59), 26–34 years for cancer of the cervix uteri (median 28) and 24–38 years for HD (median 28). SIRs were somewhat more marked in the 15–34 years age group for cancer of the cervix and HD, and among intravenous drug users (IDUs) for cancer of the cervix (Table 2).

Finally, in the same dataset, 111 cases of NHL (SIR 58.6, 95% CI 48.2–70.6) and 151 of KS (SIR > 1300) were identified.

### DISCUSSION

Three cancer sites or types, other than KS and NHL, have been found in our study to be significantly increased in persons with AIDS: HD, cancer of the cervix uteri and non-melanomatous skin cancer.

HD showed a ninefold excess, which was consistent in strata of gender, age and HIV exposure category. The highest SIRs were found in the years around AIDS diagnosis, suggesting that they were proportional to the degree of immunosuppression, as for NHL (IARC, 1996). Similar results have emerged in two other linkage studies from San Francisco, USA (SIR 8.8, 95% CI 5.0–14.3) (Reynolds et al, 1993), and New South Wales, Australia (SIR 8.5, 95% CI 4.1–16) (Grulich et al, 1997). In addition, these findings add to several reports of HD excesses in HIV-seropositive cohorts (Lyter et al, 1995; Serraino et al, 1997), to the twofold increase in HD in never married men aged 25–54 in San Francisco between the periods 1973–79 and 1988–90 (Rabkin and Yellin, 1994) and in HIV-seropositive black patients in South Africa (non-significant difference) (Sitas et al, 1997). Thus, an association between HD and HIV infection seems to be well established, although with a SIR much lower than that for NHL (IARC, 1996). This is in contrast to the lack of an increase in HD in any of the studies of transplant recipients (Kinlen, 1996). B-cell hyperplasia is not a feature of iatrogenic immunosuppression as it is in HIV infection, which may explain why certain types of lymphomas, namely HD and Burkitt's lymphoma, are relatively frequent in AIDS patients but not in transplant recipients (IARC, 1996).

© Cancer Research Campaign 1998

Misdiagnosis of NHL as HD cannot be totally ruled out because the classification of lymphatic neoplasms in AIDS patients is difficult (Gaidano and Carbone, 1995). Histological confirmation was, however, present and checked for all cases of HD in our study. In fact, as in previous reports (Andrieu et al, 1993; Serraino et al, 1993) the nodular sclerosis type (the commonest type in this age group in the general population; Serraino et al, 1993) was not found; instead, tumours of mixed cellularity type predominated. Besides being more aggressive and involving bone marrow more frequently, HD in HIV-infected patients seems to be associated with Epstein–Barr virus more commonly than in the general population (Tirelli et al, 1995).

Cancer of the cervix uteri was found in four women (16 times the expected number). All cancers of the cervix had occurred several (i.e. 5, 8, 9 and 51) months before the diagnosis of AIDS. Among previous record linkage studies, only Reynolds et al (1993) reported on malignancies (only 0.3% of the total) in women with AIDS. SIR was 6.5 for in situ carcinoma of the cervix, based on two cases (Reynolds et al, 1993).

HIV-associated immunosuppression has been consistently shown to increase a person's risk of diseases caused by human papillomavirus (HPV), including squamous intra-epithelial lesions and in situ carcinoma of the cervix. However, confounding caused by sexual promiscuity has been difficult to exclude (IARC, 1995, 1996). Besides being more prevalent in HIV-seropositive women in cross-sectional studies (IARC, 1996), HPV cervical infections were shown in a prospective investigation (Sun et al, 1997) to be about seven times more likely to persist in seropositive than in HIV-seronegative women. Persistence was inversely related to $CD4^+$ counts (Sun et al, 1997). In a case–control study of black cancer patients in South Africa, HIV seropositivity was slightly lower (3.9%) among 180 women with cervical cancer than in 218 women (8.3%) with a variety of cancers not suspected of having an infectious cause (Sitas et al, 1997). Furthermore, the AIDS epidemic did not seem to affect cervical cancer incidence during the late 1980s in the USA (Rabkin et al, 1993) or in Africa (Rabkin and Blattner, 1991; Sitas et al, 1997), but various factors, including downward incidence trends in the general population, relatively late spread of AIDS among women and cervical screening practices (i.e. the frequent removal of in situ lesions), may account for the difficulty of establishing an association between HIV and invasive cervical cancer. However, the present elevated SIR for cervical cancer needs further confirmation.

For non-melanomatous skin cancer, a moderate excess was seen in persons with AIDS. Misclassification with KS seems unlikely, as all the non-melanomatous skin cancers in persons with AIDS were histologically confirmed. Of more concern is the completeness of CR data on skin cancer in the general population. Two melanomas and one cancer of the lip also exceeded the expected numbers, although the increases were not significant. No data on skin cancer from other linkage studies are available, but Grulich et al (1997) reported a fourfold increased risk of cancer of the lip. In addition, results from HIV-seropositive cohorts are scanty and inconclusive (Ragni et al, 1993; Lyter et al, 1995). It is clear, however, that skin cancer in our study affected more older individuals than were affected by HD and cancer of the cervix. Furthermore, the excess in persons with AIDS was much lower than in transplant recipients in the United Kingdom–Australasian Collaborative Study (9.1-fold increased risk; Kinlen et al, 1983) and those from Nordic countries (over 20-fold; Birkeland et al, 1995), especially for squamous cell carcinomas.

The findings for cancer of the lung and brain are of interest, but masses of non-neoplastic origin or attributable to NHL are common at AIDS diagnosis, and these may be misdiagnosed as cancers of the lung or brain, particularly as histological confirmation is often lacking. Thus, there is a need for the quality of cancer registration in AIDS patients to be improved.

No significantly elevated SIRs were found for other cancer sites or types, although several SIRs were above unity and confidence intervals were broad because of the small study size. At variance with a study from the USA (Melbye et al, 1994), no excess of anal cancer was identified. This may be because in Italy only a minority of AIDS patients are recorded as homosexual or bisexual (18% of male cases) (Dal Maso et al, 1995). Furthermore, levels of promiscuity in this group seem lower than in the USA (Franceschi et al, 1989).

Strengths and weaknesses of the study in which CRs and AIDS registries are linked have been reviewed elsewhere (Melbye et al, 1994; Coté et al, 1995; Biggar et al, 1996; Franceschi et al, 1998). We could have underestimated the number of persons with AIDS and cancer through failures of reporting to either registry or through missed linkages, although the procedures we used have been validated (Franceschi et al, 1998). The problem of migration out of CR areas should be less severe in Italy than elsewhere, as population mobility is comparatively low (Franceschi et al, 1998). Finally, SIRs of some tumours (e.g. HD) after the diagnosis of AIDS may have been increased by more thorough investigation of accompanying illnesses. However, as cancer excesses were found before AIDS diagnosis, they should not be totally attributable to the intensive evaluation of persons at the time of AIDS discovery (Biggar et al, 1996).

### ACKNOWLEDGEMENTS

This work was supported by a grant (20.A.1.1) from the Istituto Superiore di Sanità, Rome. The authors wish to thank Dr Robert Biggar, Professor Carlo La Vecchia and Dr Fabio Barbone for their useful comments and Mrs Anna Redivo for editorial assistance.

### REFERENCES

Andrieu JM, Roithmann S, Tourani JM, Levy R, Desablens B, le Maignan C, Gastaut JA, Brice P, Raphael M and Tailln B (1993) Hodgkin's disease during HIV1 infection: the French registry experience. *Ann Oncol* 4: 635–641

Biggar RJ, Rosenberg PS, Coté T and the Multistate AIDS/Cancer Match Study Group (1996) Kaposi's sarcoma and non-Hodgkin's lymphoma following the diagnosis of AIDS. *Int J Cancer* 68: 754–758

Birkeland SA, Storm HH, Lamm LU, Barlow L, Blohme I, Forsberg B, Eklund B, Fjeldborg O, Friedberg M, Frodin L, Glattre E, Halvorsen S, Holm NV, Jakobsen A, Jorgensen HE, Ladefoged J, Lindholm T, Lundgren G and Pukkala E (1995) Cancer risk after renal transplantation in the Nordic countries, 1964–1986. *Int J Cancer* 60: 183–189

Breslow NE and Day NE (1987) *Statistical methods in cancer research*. Vol. II. *The Design and Analysis of Cohort Studies*. IARC Scientific Publication no. 82, pp. 60–71. IARC: Lyon

Conti S, Farchi G, Galletti A, Masocco M, Napoli PA, Pezzotti P, Rezza G, Toccaceli V and Cariani G (1997) La notifica della mortalità per AIDS in Italia (1992): qualità della certificazione e sottonotifica. *G Ital dell'AIDS* 8: 12–16

Coté TR, O'Brien TR, Ward JW, Wilson SE, Blattner WA and the AIDS and Cancer Registry Linkage (1995) Measurement and enhancement of registry completeness. *Prev Med* 24: 375–377

Dal Maso L, Franceschi S, Negri E, Serraino D, La Vecchia C and Ancelle-Park RA (1995) Trends of AIDS incidence in Europe and the United States. *Soz Praventivmed* 40: 239–265

Franceschi S, Serraino D, Saracchini S, Vaccher E, Tirelli U and the AIDS and Related Syndromes Study Group (1989) Risk factors for human immunodeficiency virus (HIV) infection among homosexual and bisexual men

© Cancer Research Campaign 1998 

in a region at low risk for AIDS: the Northeastern part of Italy. *Rev Epidemiol Sante Publique* **37**: 103–108

Franceschi S, Dal Maso L, Arniani S, Lo Re A, Barchielli A, Milandri C, Simonato L, Vercelli M, Zanetti R, Rezza G for the Cancer and AIDS Registry Linkage Study (1998) Linkage of AIDS and cancer registries in Italy. *Int J Cancer* **75**: 831–834

Gaidano G and Carbone A (1995) AIDS-related lymphomas: from pathogenesis to pathology. *Br J Haematol* **90**: 235–243

Grulich A, Wan X, Law M, Coates M and Kaldor J (1997) Rates of non-AIDS defining cancers in people with AIDS. *J Acquired Immune Defic Syndrome Hum Retrovirol* **14**: A18

IARC Working Group on the Evaluation of Carcinogenic Risks to Humans (1995) *IARC Monograph on the Evaluation of Carcinogenic Risks for Humans from the Human Papillomaviruses*, Vol. 64. IARC: Lyon

IARC Working Group on the Evaluation of Carcinogenic Risks to Humans (1996) *IARC Monograph on the Evaluation of Carcinogenic Risks for Humans from Human Immunodeficiency Viruses and Human T-Cell Lymphotropic Viruses*. Vol. 67. IARC: Lyon

Kinlen LJ (1996) Immunologic factors, including AIDS. In: *Cancer Epidemiology and Prevention*, 2nd edn. Schottenfeld D and Fraumeni JF (eds), pp. 532–545. Oxford University Press: New York

Kinlen L, Doll R and Peto J (1983) The incidence of tumors in human transplant recipients. *Transplantation Proc* **15**: 1039–1042

Lyter DW, Bryant J, Thackeray R, Rinaldo CR and Kingsley LA (1995) Incidence of human immunodeficiency virus – related and non-related malignancies in a large cohort of homosexual men. *J Clin Oncol* **13**: 2540–2546

Melbye M, Coté TR, Kessler L, Gail M, Biggar RJ and The AIDS/Cancer Working Group (1994) High incidence of anal cancer among AIDS patients. *Lancet* **343**: 636–639

Muir C, Waterhouse J, Mack T, Powell J and Whelan S (1987) *Cancer Incidence in Five Continents*, Vol. 5. IARC Scientific Publication no. 88. IARC: Lyon

Parkin DM, Muir CS, Whelan SL, Gao YT, Ferlay J and Powell J (1992) *Cancer Incidence in Five Continents*, Vol. 6. IARC Scientific Publication no. 120. IARC: Lyon

Parkin DM, Whelan SL, Ferlay J, Raymond L and Young J (1997) *Cancer Incidence in Five Continents*, Vol. 7. IARC Scientific Publication no. 143. IARC: Lyon

Rabkin CS and Blattner WA (1991) HIV infection and cancers other than non-Hodgkin's lymphoma and Kaposi's sarcoma. *Cancer Surveys* **10**: 151–160

Rabkin CS, Biggar RJ, Baptiste MS, Abe T, Kohler BA and Nasca PC (1993) Cancer incidence trends in women at high risk of human immunodeficiency virus (HIV) infection. *Int J Cancer* **55**: 208–212

Rabkin CS and Yellin F (1994) Cancer incidence in a population with a high prevalence of infection with human immunodeficiency virus type 1. *J Natl Cancer Inst* **86**: 1711–1716

Ragni MV, Belle SH, Jaffe R, Duerstein SL, Bass DC, McMillan CW, Lovrien EW, Aledort LM, Kisker CT, Stabler SP, Hoots WK, Hilgartner MW, Cox-Gill J, Buchanan GR, Sanders NL, Brettler DB, Barron LE, Goldsmith JC, Ewenstein B, Smith KJ, Green D, Addiego JE and Kingsley LA (1993) Acquired immunodeficiency syndrome-associated non-Hodgkin's lymphomas and other malignancies in patients with hemophilia. *Blood* **81**: 1889–1897

Reynolds P, Saunders LD, Layefsky ME and Lemp GF (1993) The spectrum of acquired immunodeficiency syndrome (AIDS)-associated malignancies in San Francisco, 1980–1987. *Am J Epidemiol* **137**: 19–30

Serraino D, Carbone A, Franceschi S and Tirelli U for the Italian Cooperative Group on AIDS and Tumours (1993) Increased frequency of lymphocyte depletion and mixed cellularity subtypes of Hodgkin's disease in HIV-infected patients. *Eur J Cancer* **29A**: 1948–1950

Serraino D, Pezzotti P, Dorrucci M, Alliegro MV, Sinicco A and Rezza G (1997) Cancer incidence in a cohort of human immunodeficiency virus seroconverters. *Cancer* **79**: 1004–1008

Sitas F, Bezwoda WR, Levin V, Ruff P, Kew MC, Hale MJ, Carrara H, Beral V, Fleming G, Odes R and Weaving A (1997) Association between human immunodeficiency virus type 1 infection and cancer in the black population of Johannesburg and Soweto, South Africa. *Br J Cancer* **75**: 1704–1707

Sun X-W, Kuhn L, Ellerbrock TV, Chiasson MA, Bush TJ and Wright Jr TC (1997) Human papillomavirus infection in women infected with the human immunodeficiency virus. *N Engl J Med* **337**: 1343–1349

Tirelli U, Errante D, Dolcetti R, Gloghini A, Serraino D, Vaccher E, Franceschi S, Boiocchi M and Carbone A (1995) Hodgkin's disease and human immunodeficiency virus infection: clinicopathologic and virologic features of 114 patients from the Italian Cooperative Group on AIDS and Tumours. *J Clin Oncol* **13**: 1758–1767

World Health Organization (1977) *International Classification of Diseases*, 9th revision. WHO: Geneva

### APPENDIX: CANCER AND AIDS REGISTRY LINKAGE STUDY

Sezione IST, Firenze (M Geddes); Servizio di Epidemiologia, Centro di Riferimento Oncologico, Aviano (A Lo Re); Registro Tumori della Provincia di Ferrara (S Ferretti, S Zago); Registro Tumori Toscano, Firenze (D Balzi); Registro Tumori di Popolazione della Provincia di Latina (M Caperle, V Ramazzotti); Registro Tumori Ligure c/o IST, Genova (MA Orengo); Registro Tumori della Romagna, Forlì (C Milandri); Registro Tumori Lombardia c/o INT, Milano (G Tagliabue); Registro Tumori del Veneto, Padova (S Guzzinati); Registro Tumori Provincia di Macerata, Camerino (F Pannelli, S Vitarelli); Registro Tumori di Modena (M Federico, L Mangone); Registro Tumori della Provincia di Parma (V De Lisi, L Serventi); Registro Tumori Ragusa (L Gafà, R Tumino); Registro Tumori Piemonte, Torino (S Rosso); Registro dei Tumori della Provincia di Trieste (G Stanta, F Cavallieri); Registro dei Tumori Infantili del Piemonte, Torino (C Magnani); Istituto Superiore di Sanità, Roma (P Pezzotti).

© Cancer Research Campaign 1998